# Supplementary material for: Obstacles to inclusion and threats to civil rights: An integrative review of the social experiences of service dog partners in the United States
Source: PLoS One. 2025 Mar 19;20(3):e0313864. doi: 10.1371/journal.pone.0313864 (PMC11922260; doi:10.1371/journal.pone.0313864)
Supplement: S2 File — (DOCX) [file pone.0313864.s002.docx]

**Appendix 1. Database Search Vocabulary and Syntax Example: PubMed**

| **PubMed Search Vocabulary and Syntax** |
| --- |
| ("service animals"[Title/Abstract] OR "service animal"[Title/Abstract] OR "service dogs"[Title/Abstract] OR "service dog"[Title/Abstract] OR "assistance animal"[Title/Abstract] OR "assistance animals"[Title/Abstract] OR "assistance dog"[Title/Abstract] OR "assistance dogs"[Title/Abstract]) AND ("civil rights"[Title/Abstract] OR "access denial"[Title/Abstract] OR “inclusion”[Title/Abstract] OR “exclusion”[Title/Abstract] OR “stigma”[Title/Abstract] OR “discrimination”[Title/Abstract] OR “attitudes”[Title/Abstract] OR “prejudice”[Title/Abstract] OR "social integration"[Title/Abstract] OR "social support"[Title/Abstract] OR "social participation"[Title/Abstract] OR "social connection"[Title/Abstract] OR “loneliness”[Title/Abstract] OR "social isolation"[Title/Abstract]) |

**Appendix 2. Methodological Rigor Scoring**

| Empirical (All)^1-3^ |
| --- |
| Was an aim, purpose, objective, or research question of the study stated? |
| Is there a clear description of eligibility (inclusion/exclusion) criteria of participants? |
| Was ethical approval sought and received, and clearly stated including source? |
| Were study participant's disabilities independently assessed by researcher? |
| Were characteristics of the service animals in the study described, including their provider and training? |
| If participants have had service animals for variable amounts of time, was time since placement considered for analyses? |
| Were limitations of the study discussed in detail, taking into account sources of potential bias or imprecision? |
| Empirical (Quantitative)^1-3^ |
| Were hypotheses or specific aims stated? |
| Are effect sizes for most outcomes provided? |
| Does the design include a control condition? |
| Does the study provide estimates of the variability in the data for most outcomes? |
| Have actual probability values been reported for most outcomes? |
| Was there a demonstration that groups or baseline characteristics were comparable on demographic and medical variables? |
| Were key demographic characteristics of study participants described including average age and percent male/female? |
| Were statistical values for most outcomes reported? |
| Empirical (Qualitative)^1-3^ |
| Are negative/discrepant results taken into account? |
| Are sequences from the original data presented and were these fairly selected? |
| Are the explanations for the results plausible and coherent? |
| Do the authors report achieving data saturation? |
| Is it clear how the themes and concepts were identified in the data? |
| Is it clear what methods were used to collect data with sufficient details, including type of method and tools? |
| Is there triangulation of data? |
| Did more than one researcher perform the analysis? |
|  |
| Theoretical^4^ |
| Is the aim, purpose, or objective for the development of the theory stated? |
| Is there a clear description of the theory’s meaning, concepts/statements, definitions, and use? |
| Is it demonstrated that the theory can make accurate predictions? |
| Is the theory useful or helpful in understanding or predicting outcomes and generating research studies? |
| Is the theory generalizable or transferable (i.e., can it be applied to persons similar to those actually studied)? |
| Is the theory parsimonious (i.e., are complex phenomena explained simply or briefly while being complete in their explanation)? |
| Is the theory testable? |
|  |
| Law Review^5-7^ |
| Is the law review article published with an American Bar Association (ABA)-accredited school? |
| Does the primary author have a JD, JsD, or PhD? |
| Does the primary author hold an academic position (i.e., associate professor or professor)? |
| Was the publishing school’s rank in the Top 100? |
| Does the law review's introduction include a thesis statement with a claim that is either descriptive or prescriptive in nature? |
| Is there a background section following the introduction which summarizes the necessary facts? |
| Does the argument apply to more than one jurisdiction? |
| Is there a proposed solution relevant to the topic? |
| Does the law review recall and synthesize legal precedents that add to the author's reasoning? |
| Is there a discussion of the positive practical consequences of the proposed solution? |
| Does the law review include an acknowledgment of uncertainty or opposing viewpoints? |

**Risk of Bias Question Sources**

1. LaB NioHNH. (2013). *Study Quality Assessment Tools*. Retrieved November 17, 2021, from https://www.nhlbi.nih.gov/health-topics/study-quality-assessment-tools
2. Schulz, K. F., Altman, D. G., Moher, D., & the CONSORT Group. (2010). CONSORT 2010 Statement: Updated guidelines for reporting parallel group randomised trials. *BMC Medicine*, *8*(1), 18. https://doi.org/10.1186/1741-7015-8-18
3. Specialist Unit for Review Evidence. (n.d.). *Critical appraisal tools*. Cardiff University. Retrieved November 17, 2021, from https://www.cardiff.ac.uk/specialist-unit-for-review-evidence/resources/critical-appraisal-checklists
4. Toronto, C. E., & Remington, R. (2020). *A step-by-step guide to conducting an integrative review*. Springer.
5. U.S. News. (2023). *Best Law Schools*. 2023-2024 Best Law Schools. https://www.usnews.com/best-graduate-schools/top-law-schools/law-rankings
6. von Elm, E., Altman, D. G., Egger, M., Pocock, S. J., Gøtzsche, P. C., & Vandenbroucke, J. P. (2014). The Strengthening the Reporting of Observational Studies in Epidemiology (STROBE) Statement: Guidelines for reporting observational studies. *International Journal of Surgery*, *12*(12), 1495–1499. https://doi.org/10.1016/j.ijsu.2014.07.013
7. Volokh, E. (2016). *Academic legal writing: Law review articles, student notes, seminar papers, and getting on law review* (5th ed.). Foundation Press. https://www.westacademic.com/Volokhs-Academic-Legal-Writing-Law-Review-Articles-Student-Notes-Seminar-Papers-and-G-9781634598880
