# Supplementary material for: Obstacles to inclusion and threats to civil rights: An integrative review of the social experiences of service dog partners in the United States
Source: PLoS One. 2025 Mar 19;20(3):e0313864. doi: 10.1371/journal.pone.0313864 (PMC11922260; doi:10.1371/journal.pone.0313864)
Supplement: Attachment — Submitted filename: ResponseToReviewers_240813.pdf [file pone.0313864.s003.pdf]

Dear Reviewers,

Thank you very much for your time and expertise in reviewing our manuscript. We are grateful for your suggestions which we believe have strengthened the quality of our manuscript. Please find the itemized responses to each of your comments below, in addition to an updated manuscript using track changes. In our responses below, bold emphasis indicates new or modified information.

Reviewer #1:

Overall, this is an excellent manuscript and very well-written. It is quite lengthy, but I'm not sure if that needs to change because everything you've included is well-justified. I do wonder about your intended audience, though. I believe this article meets all of the journal publication requirements. I have provided additional points of feedback below.

> Thank you for this kind feedback. Our intended audience is broad-ranging; we believe this information is relevant to everyone including researchers, policymakers, service dog trainers and organizations, members of the public, and even service dog handlers themselves.

Title: given the exclusive focus on American-based research, this manuscript title would benefit from reflecting this in some way.

> We have added United States to the title. It now reads: "Obstacles to inclusion and threats to civil rights: An integrative review of the social experiences of service dog partners **in the United States**."

Lines 76-79: "For example, service animals may lawfully be denied entry in spaces where their presence would fundamentally alter the business or entity, such as sterile environments in a hospital or certain parts of a zoo wherein seeing or smelling a service animal could disrupt the resident animals." – It would be helpful to include the legislation code here or a citation.

> Thank you for the suggestion, we have added the legislation code (28 C.F.R. § 36.302).

Lines 83-85: "Although legislative recognition of service animals in the United States is relatively recent, training service animals – specifically, guide dogs – can be traced back to late 18th century France." – Given the myriad terms, it would be helpful to explain somewhere exactly which categories fall under "service dog", beyond just guide dogs. Also, how do "assistance dogs" relate? I see mention later on of service and emotional support animals falling under the broader definition of "assistance animals" except in other publications service and assistance animals are considered synonyms.

> We strongly agree that the terminology adds to the confusion around this topic. We have revised the sentence in question to remove the not-yet-defined term. It now reads "Although legislative recognition of service animals in the United States is relatively recent, **their** training can be traced back to late 18th-century France."
>
> Additionally, we have added a new table to the introduction to help clarify this and have copied it below for ease of reference:

Table 1. Terminology

Umbrella Terms	Commonly Understood Definition
Service animal<br>Service dog	A dog (or miniature horse) trained to do work or perform tasks for a person with a disability.<br>***Most common umbrella term in the US, as defined by the ADA.***
Assistance animal<br>Assistance dog	Internationally, this term is equivalent to service animal or service dog and is the most common umbrella term.<br>In the US, in certain situations such as housing, may also include emotional support animals (not trained in specific work or tasks).

Role-Specific Terms	Commonly Understood Definition
Guide dog	Service/assistance dog trained to help a person who is blind navigate their environment.
Hearing dog	Service/assistance dog trained to help a person who is d/Deaf or hard of hearing by alerting them to important sounds.
Mobility dog	Service/assistance dog trained to help somebody with a mobility-related disability, for example, a person who uses a wheelchair.
Medical alert dog	Service/assistance dog trained to alert or respond to the onset of a medical event (such as a seizure or high/low blood sugar).
Psychiatric service dog	Service/assistance dog trained to help a person with a psychiatric disability or neurodevelopmental disorder.

Lines 85-86: "Nowadays, there are tens of thousands of human-service dog dyads ("teams") both nationally and globally (10,11)": You have only sited ADI and IGDF who solely publish on their membership data. As such, this estimate may not be wholly accurate. I believe a comment on this should be included.

> Thank you for pointing out this omission. The sentence now reads: "Nowadays, there are tens of thousands of human-service dog dyads ("teams") **reported through accrediting entities** nationally and globally (10,11), **and an additional unknown number of human-service dog teams trained outside of accredited schools**.

Lines 132-136: How does your article differ from others on this topic? Has anything like this been published before? How does your research uniquely contribute to this area? The word "impact" implies causality, and many of the articles you extracted do not examine causal links. Given the methods and results of your article, your research questions do not fully reflect what you examined and found. I would recommend clarifying that you questioned: "What are the 'reported/published' social effects faced by….."

> We have revised these sentences as follows: "Research on the social **experiences** of service dog partners remains limited; **to our knowledge, no comprehensive review has been published on this topic.** … Our research question is: what are the social **experiences reported by** service dog partners …"
>
> We have also made an effort to change the "impact" wording throughout the paper.

Methods: Is this type of research exempt from review with your institutional research ethics board?

Yes, and we have added a sentence to the methods to specify this ("**Institutional Review Board approval was not required as the research did not involve human subjects.**")

Line 145: "(3) population comprises individuals with disabilities in the United States" - are disabilities self-determined or do they need to be medically determined?

We did not specify this, and the articles included both. We have clarified the inclusion criteria to read "… individuals with disabilities **(self-reported or diagnosed)** in the …"

Lines 159-160: Inclusion criteria included "service miniature horse", so why was this not a search term?

Thank you for asking about this. This was an unintentional omission. However, the terms service animal(s) and assistance animal(s) should have been sufficient to capture literature on service miniature horses, had there been any.

Lines 202-204: Inclusion criteria included "service miniature horse", so why were only the social impacts of service dog partners analyzed?

No articles reported information about service miniature horses, only dogs. However, we appreciate the point that because this is the methods section, we should use the broader term and have thus revised this to read "…social **experiences reported by** service **animal** partners…"

Lines 284-286: Regarding the definitions of stigma and discrimination you adopted, do these reflect the published literature and consensus on these constructs?

These definitions are based on the American Psychological Association (APA) Dictionary of Psychology, and we have added these citations:

American Psychological Association. APA Dictionary of Psychology: Stigma (n.). https://dictionary.apa.org/stigma, 2024.
American Psychological Association. APA Dictionary of Psychology: Discrimination (n.). https://dictionary.apa.org/discrimination, 2024.

Lines 520-522: Can you please clarify what you mean by "…study participants of the reviewed articles recommended…" – do you mean you're reporting on what past study participants suggested in the articles you examined? Or did you somehow contact past study participants to get their views?

Thank you for clarifying this; we intended to report on what past study participants suggested in the articles we examined. We have reworded to: "**Based on the reviewed articles, several solutions have been** recommended…"

Reviewer #2:
This is a comprehensive, well-done review of the social experiences of service dog partners. To improve readability it is suggested that sub-headings be added to the Discussion section of the manuscript.

Thank you for the kind feedback. We have added sub-headings to the Discussion section.

If available, quotes from service dog partners may enhance the impact of the findings. These could either be in the literature review section or in a table.

> This is a great suggestion. We selected some exemplar quotes and incorporated them into a figure to enhance impact without unduly adding to the paper's length.
